# Complementary and alternative medicine in the management of hypertension in an urban Nigerian community

**DOI:** 10.1186/1472-6882-10-36

**Published:** 2010-07-19

**Authors:** Pauline E Osamor, Bernard E Owumi

**Affiliations:** 1 Department of Sociology, Bowen University, Iwo, Nigeria and Department of Sociology, University of Ibadan, Ibadan, Nigeria

## Abstract

**Background:**

Hypertension is a common non communicable condition worldwide. In developing countries (including Nigeria), the use of complementary and alternative medicine (CAM) is common. This study investigated the frequency and factors associated with use of CAM among hypertensive subjects in an urban Nigerian community. Perspectives about the management of hypertension were obtained from CAM practitioners in the community.

**Methods:**

Four hundred and forty hypertensive subjects in Idikan community, Ibadan, were interviewed using a semi-structured survey instrument. Association between categorical variables was tested using the chi-square test. Logistic regression analysis was done to identify independent predictor variables of CAM use, with CAM use as the outcome variable and the demographic and belief items as predictor variables. In-depth interviews were conducted with all known CAM practitioners in the community on issues relating to their beliefs, knowledge, practice and experiences in managing patients with hypertension in the community.

**Results:**

In the study sample, 29% used CAM in the management of their hypertension. Among those using CAM, the most common forms used were herbs (63%) and garlic (21%). Logistic regression analysis revealed that four variables were independent predictors of CAM use: being male (OR 2.58, p < 0.0001), belief in supernatural causes of hypertension (OR 2.11, p = 0.012), lack of belief that hypertension is preventable (OR 0.57, p = 0.014) and having a family history of hypertension (OR1.78, p = 0.042). Other factors such as age, educational level and occupation were not independent predictors of CAM use. Interviews with CAM practitioners revealed that they believed hypertension was caused by evil forces, stress or "too much blood in the body". They also thought they could cure hypertension but that reduced costs (compared to hospitals) was one of the reasons most of their clients consult them.

**Conclusions:**

The use of CAM is common among hypertensive subjects in this urban Nigerian community. Men were more than twice as likely to use CAM and belief in supernatural causes of hypertension was the most notable belief predicting CAM use. Interviews with CAM practitioners yielded useful perspectives about the role they play in hypertension management in the community. This study adds to the small but growing literature about the use of CAM in hypertension in sub Saharan Africa. Further studies in hypertension and other non communicable disease are needed.

## Background

Hypertension is one of the most common non-communicable diseases worldwide affecting up to 800 million (or 20%) of the world's adult population [1]. It is estimated to cause 4.5% of the current global disease burden and is often as prevalent in many developing countries as in developed countries [2]. While reliable data from large scale, population based studies in sub-Saharan Africa are few, the evidence suggests that overall hypertension prevalence is between 10-15%, with specific settings (for example, some urban centers) often having twice these prevalence rates [3-5]. More importantly, hypertension awareness, treatment and control are quite poor in sub-Saharan Africa [3,6]. Multiple social, economic, infrastructural and cultural factors contribute to this state of affairs [4,6,3].

Health seeking and utilization of health care services for hypertension in developing countries is often a complex issue, since people often seek care from multiple sources outside the formal orthodox health care system. Empirical studies of preventive and curative service have often found that the use of health care services in general is related to availability, quality and cost of services as well as to social structure, health beliefs and personal characteristics of the user [7]. The frequency of utilization of CAM is increasing worldwide, and is well documented in both African and other global populations to be between 20 - 80% [8-10]. Traditional health practitioners often play a major role in health care in many countries [11]. For example, the reported use of herbal medicine (a form of CAM) in the general population from different parts of the world varies, with figures like 40% in the United States, 38.5% among the Indian community of Chatsworth in South Africa [12] and 48.5% in Australia [13]. For hypertension specifically, Shafig and colleagues [14] reported that as many as 63.9% of their hypertensive subjects in a clinic in India took herbal medicines, while in Morocco 80% of patients with hypertension and diabetes used medicine plants to treat their ailments [8]. In Nigeria, two hospital-based studies found that herbal medicine among hypertension patients was 39% and 24%, respectively [15,16]. Therefore, in common with such obviously life-threatening and severe conditions as cancer in which CAM use has been well-documented [17,18]), the use of CAM in hypertension is increasingly being documented and studied [19]

The reason why patients choose to use CAM have been much discussed, but not fully understood [20,21]. The common determinants of CAM use include socio- demographic characteristics of patients. There are other complex psycho-social and cultural factors. Patients may choose to use CAM because they are dissatisfied with conventional treatments that are perceived to be ineffective or have unpleasant side effects [22,23]. Patients may also find CAM attractive because it is consonant with their personal values, religious and health philosophies [24-27]. As the orthodox medical facilities co-exists with traditional medicine systems in many regions of Africa and elsewhere, people may use medicine from one system exclusively or they may acquire medicine from each health system and use it simultaneously or sequentially. King and Homsy noted this kind of medical pluralism among patients in sub-Saharan Africa [28]. In Nigeria, multiple channels of care are utilized and range across traditional healers, spiritual churches, pharmacies [29].

The present study has the primary objective of investigating the role of complementary and alternative medicine in the management of hypertension in an urban community in south west Nigeria. Firstly, a survey of hypertensive subjects about their use and reasons for using CAM was conducted. Secondly, in-depth interviews were conducted with CAM practitioners in the community to obtain perspectives on their management of hypertension. The findings extend the scanty published work in CAM and hypertension care in Nigeria, while providing a number of interesting novel insights.

## Methods

This study was conducted in Idikan community, Ibadan, a city in the south western part of Nigeria, as part of a larger community based study of the sociological aspects of hypertension. Ethical approval for the study was obtained from the Joint University of Ibadan-University College Hospital Ethical Committee. The total population of Idikan is 7,883 [30]. Health facilities in the community include an outreach clinic run by the Department of Community Health of the University of Ibadan, a small dental clinic run by the Dental Centre of University College Hospital (UCH) and private clinics. There are also registered patent medicine stores (pharmacies) and traditional healing homes in the area, all of which are accessible to members of the community.

The study comprised two components: a quantitative study of hypertensive subjects in the community and a qualitative study of CAM practitioners practising in the community. The subjects for the quantitative study were adults (above 25 years of age) residents of Idikan who are known to have hypertension. Previous studies in the community had conducted household screening for hypertension, which facilitated the identification of hypertensive subjects in the community. The subjects for this study were selected from a list of known hypertensive subjects residing in the community that was developed from one such previous hypertension study [31] and updated for the present study during home visits. Four hundred and forty hypertensive subjects were enrolled using a consecutive sampling method. After obtaining informed consent, subjects were administered a semi-structured questionnaire that had items on several issues, including health care seeking for their hypertension, the use of CAM, what forms of CAM they used and their beliefs about the cause of hypertension.

The subjects of the qualitative study were all CAM practitioners (called indigenous healers or traditional healers) in the community who were studied using an in depth interview method. There are five traditional healing homes in the community and the healer that runs each of these homes were all interviewed. The primary goal of the interviews was to capture the observations of the CAM practitioners as they have interacted with and cared for people with hypertension in the community. The interviews also covered such issues as the respondent's perspectives of hypertension in the community, how commonly they are approached to treat hypertension or its complications and recurrent issues they have with patients who are hypertensive. Each interview lasted for between 45-60 minutes. The interviews were recorded on audio cassettes and subsequently transcribed, translated and back-translated. The final interview text was word processed for content analysis.

Data management and analysis of the survey questionnaire data was done using SPSS version 14 (SPSS Inc, Chicago, USA). Frequencies of the responses to the questions were computed and presented as percentages. Association between categorical variables was tested using the chi-square test. The belief items with responses of "Yes", "No" and "Don't know" were tested for association with CAM use in two ways: first, with all three responses (i.e. a 3 × 2 contigency table) and second, with the responses collapsed into "Yes" and "Other responses" (i.e. a 2 × 2 contigency table). To identify independent predictor variables of CAM use, a logistic regression analysis was carried out with CAM use as the outcome variable and the demographic (Table 1) and belief (Tables 2 and 3) items as predictor variables. Starting with the full model, a backward selection strategy was used to identify variables that remained in the model with a p < 0.05. The qualitative data (from the key informant interviews with CAM practitioners) was analyzed for themes about their beliefs about the causes, severity and prevalence of hypertension, why they think hypertensive patients use CAM and their experience with treating hypertensive patients with CAM. Computer assisted qualitative data analysis (CAQDAS) package Atlas/ti was used to analyze the qualitative data. Verbatim quotations that illustrated or exemplified themes are presented.

**Table 1:** Care seeking at CAM practitioner and Use of CAM to treat hypertension among 440 hypertensive subjects

		**Use of CAM**		
		**Yes**	**No**	**Total**
**Care seeking at CAM practitioner**	**Yes**	57 (13.0%)	8 (1.8%)	65
	**No**	71 (16.1%)	304 (69.1%)	375
	**Total**	128	312	440

**Table 2:** Use of CAM by demographic characteristics of participants

Characteristic	No (%)	% Those using CAM (N = 128)	% Those not using CAM (N = 312)	P*
**Age**				
Mean (SD)	59.7 (11.7)	58.6 (10.6)	60.1 (12.1)	0.211
**Sex**				
Male	153 (34.8)	51.6	27.9	
Female	287 (65.2)	48.4	72.1	<0.0001^+^
**Marital status**				
Single	9 (2.1)	2.3	1.9	
Married	312 (70.9)	80.5	67.0	
Widowed	108 (24.6)	14.1	28.9	
Divorced/Separated	11 (2.5)	3.1	2.2	0.015^+^
Religion**				
Islam	270 (61.4)	66.4	59.3	
Christianity	169 (38.4)	33.6	40.4	0.176
**Educational Level**				
No formal Education	225 (51.1)	52.3	50.6	
Primary	86 (19.5)	17.2	20.8	
Secondary	49 (11.1)	10.2	11.2	
Post Secondary	77 (17.5)	19.5	16.7	
Others (Arabic school)	3 (0.7)	0.8	0.6	0.500
**Occupation**				
Trading	220 (50.0)	43.0	52.9	
Artisan	49 (11.1)	14.8	9.6	
Teaching/Civil Servant	43 (9.8)	10.2	8.7	
Retired/not working	113 (25.7)	25.0	26.0	
Religious Teachers	15 (3.4)	7.0	2.8	0.029^+^

*P value for chi-square test, except for age (Student's t test)

**Excludes 1 person practising neither religion

^+ ^p < 0.05

**Table 3:** Beliefs about cause of hypertension by use of CAM

Perceived Cause	Response	% Those using CAM (N = 128)	% Those not using CAM (N = 312)	P*	P for Yes vs. "other"
Being born with HTN	Yes	17.2	9.9	0.066	0.034
	No	67.2	68.9		
	Don't know	15.6	21.2		
Being fat	Yes	20.3	23.4	0.168	0.482
	No	63.3	53.9		
	Don't know	16.4	22.8		
Stress	Yes	92.2	93.3	0.197	0.687
	No	6.3	3.2		
	Don't know	1.6	3.5		
Eating too much salt	Yes	18.0	17.6	0.004**	0.932
	No	64.1	49.4		
	Don't know	18.0	33.0		
Anxiety	Yes	94.5	94.6	0.997	0.993
	No	2.3	2.2		
	Don't know	3.1	3.2		
Unhappiness	Yes	89.1	90.1	0.478	0.753
	No	6.3	3.9		
	Don't know	4.7	6.1		
Pregnancy	Yes	14.1	19.9	0.346	0.151
	No	41.4	39.7		
	Don't know	44.5	40.4		
Supernatural causes	Yes	21.1	10.6	0.013**	0.004
	No	45.3	49.4		
	Don't know	33.6	40.1		
Bad luck	Yes	15.6	12.2	0.466	0.332
	No	50.8	49.0		
	Don't know	33.6	38.8		

*p value for chi-square test (3 × 2 contigency tables)

**P < 0.05

## Results

### Quantitative study

#### Characteristics of study sample

A total number of 440 respondents were studied, comprising 287 women and 153 men. The ages of respondents ranged from 25 to 90 years, with a mean of 60 (SD 12) years. Most of the respondents (71%) were married, while 25%, 2%, 2% and 0.5% were widowed, single, divorced and separated, respectively. Majority (61.4%) of respondents were Moslems, 38.4% were Christians and 0.7% was a traditional religion adherent. Respondents with no formal education constituted the highest cluster, representing 51.1% of the total respondents. Those with only primary education constituted 19.5% while those with higher national diploma or bachelor's degree constituted 17.5% and 11.1% for those with secondary school level. However, those with other types of education like Arabic school constituted only 0.7%. Occupationally, majority (50%) of the respondents were traders, while those who have retired and not working constituted 25.7%.

#### Health care seeking and Frequency of use of CAM

**Figure 1:**
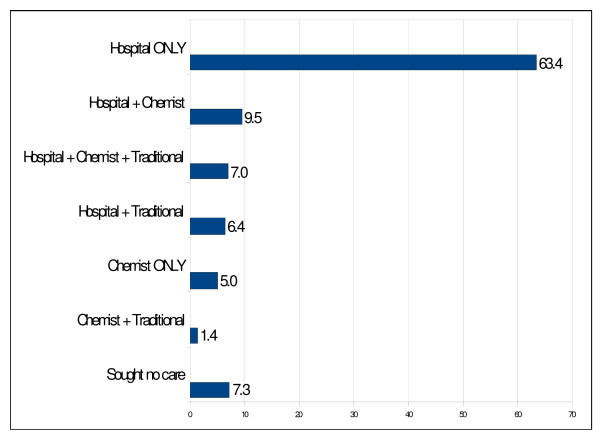
**Care-seeking behaviour for hypertension treatment**.

Figure 1 illustrates the different health facilities or places where the study participants sought health care. A large proportion (63.4%) of the respondents reported that they sought care for their condition from the hospital (the nearby University College Hospital (UCH), community health centre and private hospital); while 5% said they go to the chemist or Patent Medicine Vendor (PMV). It was interesting to note that a significant proportion of respondents used a combination of these facilities. Despite the fact that none of the respondent reported using traditional healer exclusively, 9.5% of the respondents who visited the hospital still made use of traditional medicine, while 7.3% used the chemist and traditional medicine.

Nearly one-third (29.1%) of respondents reported using CAM. About 13% of the overall sample used CAM and sought care with a CAM practitioner while 16.1% used CAM but did not seek care with a CAM practitioner (Table 1). Among those reporting use of CAM, the main forms used were: herbs only (63%), garlic (21%), and herbs and prayer (8%).

#### Factors associated with CAM use

The association of various demographic factors with CAM use was initially investigated using bivariate analysis (Table 2). Among those using CAM, 52% were men compared to 28% among those not using CAM (p < 0.0001). Similarly, marital status was significantly associated with CAM use (p = 0.015) with married people comprising 81% of those using CAM versus 67% among those not using CAM. Occupation was also associated with CAM use (p = 0.029), with a lower proportion of traders but a higher proportion of artisans among those using CAM (Table 2). Age, religion and educational level were not associated with CAM use. As shown in Table 3, belief in supernatural causes (witches, wizards) of hypertension was significantly associated with CAM use, with 21% of those using CAM believing in supernatural causes of CAM in contrast to 11% of those not using CAM (p = 0.013). Having a family member with hypertension was significantly associated with CAM use, with 23% of those using CAM having a family history of hypertension versus 14% of those not using CAM (p = 0.041). Beliefs about if hypertension was preventable, curable or can lead to complications were not associated with CAM use (Table 4).

**Table 4:** Use of CAM and other beliefs about hypertension

Belief about hypertension	Response	% Those using CAM (N = 128)	% Those not using CAM (N = 312)	P*	P for Yes vs. "other"
Hypertension is **preventable**	Yes	59.4	69.6	0.057	0.040**
	No	7.0	3.2		
	Don't know	33.6	27.2`		
Hypertension is **curable**	Yes	75.0	72.4	0.218	0.581
	No	9.4	6.1		
	Don't know	15.6	21.5		
Hypertension is **serious**	Yes	89.8	92.0	0.606	0.467
	No	3.9	2.2		
	Don't know	6.3	5.8		
Hypertension **can lead to complications**	Yes	88.3	90.4	0.631	0.508
	No	0.8	1.3		
	Don't know	10.9	8.3		
Hypertension **can be inherited or runs in families**	Yes	19.5	17.6	0.279	0.638
	No	45.3	53.5		
	Don't know	35.2	28.9		
Has family history of hypertension	Yes	22.7	14.1	0.042**	0.028**
	No	68.0	79.2		
	Don't know	9.4	6.7		

*p value for chi-square test (for 3 × 2 contigency table)

**P < 0.05

Logistic regression analysis with backward selection of variables revealed that four variables were independent predictors of CAM use: Being male, belief in supernatural causes of hypertension, lack of belief that hypertension is preventable and having a family history of hypertension (Table 5). Men were more than twice as likely to use CAM (OR 2.58, p < 0.0001) and those believing in supernatural causes of hypertension were twice as likely to use CAM (OR 2.11, p = 0.012). The other seventeen variables (including factors that were significant on bivariate analysis, such as marital status and occupation) were no longer significant predictors of CAM use once these four variables are taken into account.

**Table 5:** Logistic regression analysis to identify independent predictors of CAM use

Variable	OR	95% CI	P
Sex	2.58	1.66, 3.99	<0.0001
Belief: Supernatural causes	2.11	1.18, 3.78	0.012
Belief: Hypertension is preventable	0.57	0.36, 0.89	0.014
Has family history of hypertension	1.78	1.02, 3.10	0.042

Note: Sex coded as "Female = 0, Male = 1", other variables coded as "No = 0, Yes = 1"

Model likelihood ratio X^2 ^= 36.63, p < 0.0001

### Qualitative Study

#### CAM practitioners' views about causes, severity and prevalence of hypertension

The CAM practitioners related the cause of hypertension to being a spiritual problem, caused by evil forces and too much blood in the body. Hypertension was also seen as a poor man's sickness. Three quotes that illustrate these points are below.

"Hypertension is poor man's sickness. The stress in people's lives is too much; people are fighting and quarrelling with each other and placing cause on them or using witchcraft. All these are the cause of this disease that western medicine cannot cure and it is in our blood and body"

"Hypertension is not caused by thinking and stress like some of our customers come to tell us. If it is, why is it not affecting everybody or are they the only ones that have problems and thinking? Hypertension is a spiritual thing. It is caused by evil forces attacking people's life and health"

"Hypertension is caused by too much blood in the body. That is why they call it high blood pressure sometimes. This causes the patient to always have headache and make the heart to beat too fast. It is this too much blood that sometimes make people to paralyze and unable to talk or walk. It is a serious illness and only western medicine cannot treat it"

The interviews with the CAM practitioners were quite revealing regarding the curability of hypertension. Two of them believe that hypertension is curable and they themselves can actually cure hypertension with the herbs and concoctions they give to their clients.

"I can cure hypertension. Some of the people I have been treating are well and do not come back. When you use herbs and traditional medicine, it will go and not come back. Those that die are the ones that do not follow our instructions"

"Hypertension is curable. It is like every other sickness that you take medicine and you are cured, and it can reoccur later. For example, if you have malaria and you take iba medicine, it will go. Does that mean that person will not have malaria again? That is what I am saying. It is the same thing with hypertension. It can be cured"

The one CAM practitioner interviewed who was not sure if the disease is curable said he had seen a lot of people over the years that had been using a combination of orthodox medicine and traditional medicine, and yet remained hypertensive. He said:

"I really do not know if we can cure hypertension because there are some people that have been taking English and traditional medicine for years and they are still taking it. They still come to us to complain and some are dead. So I do not know if the sickness is cured. We just give them medicine to help them reduce the sickness and make them feel better for sometime"

#### CAM practitioners perspectives of why hypertensive patients seek CAM

The CAM practitioners stated that many people do not like going to the hospital because it is expensive and time-consuming to see the doctor. Even when they receive their drug prescriptions, they cannot afford to buy all the drugs on the prescription. Therefore, they prefer to go to the traditional or CAM practitioner. A quote from a CAM practitioner summarizes why they believe the hypertensive people in their community come to them for care is this:

*"Many of our customers come to us for help. In fact, from my records, I have about 12 customers that I am currently treating on hypertension. Majority of them come to us when they are sick and cannot afford to go to UCH. They prefer coming to us because we are within the community and we are easy to reach"*.

The issue about affording the cost of Western (orthodox) medication came up several times in each interview, with the CAM practitioners saying they offer the advantages of lower costs, ability to extend credit and allowing patients to pay in instalments, when compared with hospitals and clinics.

*"Since the research where they were giving people free drugs finished, it is difficult for people to go to the hospital, hence they come to us. Even if they do not have money, we give them medicine and they can always come back to pay later. After all, we are all in this community and we know ourselves"*.

*"The reason many people do not keep to what the doctors tell them is because it is not easy to be taking medicine every day and yet the illness is not going away. That is what our customers tell us and that is the reason they come to us. We can give them medicine on credit and they do not have to be using it everyday"*.

*"Some of these people do not like going to the hospital because there is too much wahala [*too much trouble]*. It takes time to see the doctor and when they write drugs for them, it is not in the hospital and when they go out to buy, it is more expensive. Hence they sometimes come to us because we can treat on credit. No hospital will take credit"*.

#### CAM practitioners' experiences with managing hypertension

The CAM practitioners believed that their patients complied with the treatment they prescribed for them, noting that they believe the patients also used medication from hospital as well. As one of them said:

*"If they are not taking the medicine we give them, they will not come back to say that they feel better and collect another. I think they use it together with the ones they collect from the hospital"*.

One of the interesting issues that emerged is that the CAM practitioners do not see themselves as exclusive health care providers for their hypertensive patients but, in fact, refer the patients to the hospital or health center for specific care issues like measurement of blood pressure. One practitioner said one situation where he would refer his patients to the hospital is when they are too ill:

*"When they are too sick, I ask them to go to the hospital so that nobody will die in my place. But when they can still walk around, I do not refer them or send them away"*.

## Discussion

Hypertension is a condition of sustained high blood pressure and its medical diagnosis is made after blood pressure measurements that meet the criteria for the condition. However, instead of being presented to health professionals, many symptoms are ignored, tolerated or self treated: a phenomenon known as the "symptom iceberg" [32]. Health seeking is worse for illnesses that are asymptomatic like hypertension. A feature of contemporary health care is the diverse sources of help available. For example, an individual who feel unwell may consider contacting a hospital (formal or private), over-the-counter consultation (patent medicine vendors), traditional healers or do nothing at all. In Nigeria, and in other developing countries, channels of care which are utilized are more varied, consisting of traditional healers, spiritual churches and pharmacies, among others [29]. However, even in industrialized countries having well developed systems of health care delivery, the use of sources of CAM is prevalent. For example, a recent study [33], of over 1,000 adults in Britain showed that 32% used an over-the-counter product or previously prescribed medication, 9% used home remedy or alternative medicine while under half (46%) dealt with their illness by taking no action. Therefore, treatment seeking outside the formal health sector and use of CAM seems to be worldwide, only differing in such details as frequency of use, type of CAM used and patterns of usage.

The present study was designed to estimate the frequency of use of CAM specifically in the management of hypertension and to evaluate factors associated with such use. The estimate of 29% using CAM is lower than the 39% reported in a recent such Nigerian study [34] but close to the 24% found in another Nigerian study [16]. These other studies were conducted in different Nigerian cities and were entirely hospital-based, unlike the present one which was conducted in the community. Therefore, the estimates may not be directly comparable. Compared to the Nigerian study by Amira & Okubadejo [34], our study found a different distribution of the types of CAM used by respondents. It is noteworthy that among those using CAM, the frequency of use of herbs and garlic (63% and 21%, respectively) in this study were in inverse proportion (25% and 69%, respectively) when compared to the other study. This suggests that there are true differences in the pattern of CAM usage between the two studies, despite the fact that both were conducted in Nigerian cities.

In the present study, we found that gender, marital status and occupation were significantly associated with CAM use. This is quite different from what has been reported from similar studies that examined such factors [see for example, 12, 34]. On the other hand, the lack of association of CAM use with age, religion or educational status found in this study is quite similar to what these other studies reported. It should be noted that only four factors were significant independent predictors of CAM use: being male, belief in supernatural causes of hypertension, lack of belief that hypertension is preventable and having a family history of hypertension. The other seventeen variables (including factors that were significant on bivariate analysis, such as marital status and occupation) were no longer significant predictors of CAM use once these four variables are taken into account. One noteworthy area in which this study went beyond examining demographic and/or clinical correlates of CAM use is in examining the association between CAM use and beliefs of the respondents. In this study, CAM use was significantly with belief in supernatural causes of hypertension. This provides direct evidence for previously described notions about CAM use such as that CAM are perceived to work in ways that orthodox medication may not, can work for supernatural causes (unlike orthodox medication) and may more likely lead to a cure [14,15,34]. More research is needed in this area because people's behaviour is not independent of their beliefs and evidence for how these are related in CAM use would clearly be beneficial for control programmes.

The inclusion of the perspectives of CAM practitioners is a unique feature of this study. In this context, they provided rich qualitative data which complemented the findings from the hypertensive subjects. While some of the findings of this component of this study were not surprising (for example, belief in supernatural causes of hypertension or that they can cure hypertension), an interesting findings was how they perceived that CAM use reduced the financial burden of treatment of hypertension. An important influence over treatment seeking behaviour is household ability to pay for health care [35]. Other findings from a household survey conducted in Tblilsi, Georgia, reported that health care services are a financial burden. Members of the poorer households are less likely to seek care than people from more affluent households, and devote a higher share of household monthly expenditure to health care. Amod and colleagues [36] found that when rural Nepalese feel sick, they seek healthcare only when the sickness is moderate to severe. Mild illnesses are treated at home. When the villagers seek health care, they preferred to visit traditional healers first, before visiting other health workers. Thus, studies from multiple countries have documented the role of finance and ability to pay on the utilization of the formal health care system and how in some cases lead to use of CAM.

It is important to stress the relevance of traditional medicine to the majority of Nigerians. A previous study found that traditional medicine appears to be strongly considered by Nigerian hypertensive patients as the only viable alternative for a cure for hypertension [15]. While the present study does not specifically address this question, the observation that no one in this study uses CAM exclusively suggests that they do not attribute such efficacy to their use of CAM. The findings from the qualitative study suggest that the decision to use CAM practitioners is often influenced by perception of their effectiveness, barriers regarding visiting the hospital and availability of affordable medicine. In this context, it is relevant to note that another Nigerian study which specifically asked respondents for reasons for preferring herbal medicine found similar factors, including perceived failure of allopathic medicine, relatively high cost of allopathic medicine, poor accessibility to medical facilities and uncaring attitudes of hospital staff [16]. Other studies have assessed patients' perception of the therapeutic efficacy of alternative medicines and in some of these studies, more than half of these alternative medicine users perceived that CAM was responsible for some noticeable improvement in physical or psychological well being [37,38]. The present study did not specifically address this issue and this is a limitation. However, more research is needed in the areas of both subjective and objective measures of improvement achieved with using CAM, especially because most of the current claims for the efficacy (usually touted in advertisements) are not backed by any empirical data [34].

## Conclusions

This study has identified the frequency and context of CAM use among hypertensive subjects in this urban Nigerian community. CAM practitioners were not an exclusive source of health care to the study subjects but were used in addition to other sources. The CAM practitioners interviewed identify financial reasons and convenience as the main reasons they are consulted for treatment. The study adds to the small but growing literature about the use of CAM in hypertension in sub Saharan Africa. Further studies in hypertension and other non communicable disease are needed.

## Competing interests

The authors declare that they have no competing interests.

## Authors' contributions

PEO and BEO designed the study. PEO supervised field work and did the data analysis. Both authors drafted and approved the manuscript.

## Pre-publication history

The pre-publication history for this paper can be accessed here:

http://www.biomedcentral.com/1472-6882/10/36/prepub
